# A rare splice site mutation in the gene encoding glucokinase/hexokinase 4 in a patient with MODY type 2

**DOI:** 10.18699/VJ20.41-o

**Published:** 2020-05

**Authors:** D.E. Ivanoshchuk, E.V. Shakhtshneider, A.K. Ovsyannikova, S.V. Mikhailova, O.D. Rymar, V.I. Oblaukhova, A.A. Yurchenko, M.I. Voevoda

**Affiliations:** Research Institute of Internal and Preventive Medicine – Branch of the Institute of Cytology and Genetics of Siberian Branch of the Russian Academy of Sciences, Novosibirsk, Russia Institute of Cytology and Genetics of Siberian Branch of the Russian Academy of Sciences, Novosibirsk, Russia; Research Institute of Internal and Preventive Medicine – Branch of the Institute of Cytology and Genetics of Siberian Branch of the Russian Academy of Sciences, Novosibirsk, Russia; Institute of Cytology and Genetics of Siberian Branch of the Russian Academy of Sciences, Novosibirsk, Russia; Research Institute of Internal and Preventive Medicine – Branch of the Institute of Cytology and Genetics of Siberian Branch of the Russian Academy of Sciences, Novosibirsk, Russia; Institute of Cytology and Genetics of Siberian Branch of the Russian Academy of Sciences, Novosibirsk, Russia; Institute of Cytology and Genetics of Siberian Branch of the Russian Academy of Sciences, Novosibirsk, Russia; Research Institute of Internal and Preventive Medicine – Branch of the Institute of Cytology and Genetics of Siberian Branch of the Russian Academy of Sciences, Novosibirsk, Russia Institute of Cytology and Genetics of Siberian Branch of the Russian Academy of Sciences, Novosibirsk, Russia

**Keywords:** human, maturity onset diabetes of the young, MODY2, glucokinase gene, next-generation sequencing, genetic analysis, bioinformatics., человек, диабет взрослого типа у молодых, MODY2, ген глюкокиназы, секвенирование нового поколения, генетический анализ, биоинформатика

## Abstract

The article presents a variant of maturity onset diabetes of the young type 2, caused by a rare mutation in the GCK gene. Maturity onset diabetes of the young (MODY) is a hereditary form of diabetes with an autosomal dominant type of inheritance, an onset at a young age, and a primary defect in pancreatic β-cell function. This type of diabetes is different from classical types of diabetes mellitus (DM1 and DM2) in its clinical course, treatment strategies, and prognosis. Clinical manifestations of MODY are heterogeneous and may vary even among members of the same family, i. e., carriers of identical mutations. This phenotypic variation is due to the interaction of mutations with different genetic backgrounds and the influence of environmental factors (e. g., lifestyle). Using next-generation sequencing technology, the c.580–1G>A substitution (IVS5 –1G>A, rs1554335421) located in an acceptor splice site of intron 5 of the GCK gene was found in a proband. The identified variant cosegregated with a pathological phenotype in the examined family members. The GCK gene encodes glucokinase (hexokinase 4), which catalyzes the first step in a large number of glucose metabolic pathways such as glycolysis. Mutations in this gene are the cause of MODY2. The illness is characterized by an insignificant increase in the fasting glucose level, is a well-controlled disease without medication, and has a low prevalence of micro- and macrovascular complications of diabetes. The presented case of MODY2 reveals the clinical significance of a mutation in the splice site of the GCK gene. When nonclassical diabetes mellitus is being diagnosed in young people and pregnant women, genetic testing is needed to verify the diagnosis and to select the optimal treatment method. Key words: human; maturity onset diabetes of the young; MODY2; glucokinase gene; next-generation sequencing; genetic analysis; bioinformatics.

## Abstract (ru)

В статье рассмотрен вариант развития моногенной формы сахарного диабета (MODY), обуслов- ленный редкой мутацией в гене GCK. Диабет MODY представляет собой сахарный диабет с аутосомно-доми- нантным типом наследования, возникающий в молодом возрасте и проявляющийся в дисфункции β-клеток поджелудочной железы. Этот тип отличается от классических типов сахарного диабета (СД1, СД2) клиниче- ским течением, тактикой лечения и прогнозом для пациента. Клинические проявления MODY гетерогенны и могут различаться даже у представителей одной семьи, носителей одинаковых мутаций. Это обусловлено как сочетанием мутаций в различных генах у индивидуума, так и воздействием внешних факторов. Методом секвенирования нового поколения у пробанда была идентифицирована замена c.580 –1G>A (IVS5 –1G>A, rs1554335421), локализующаяся в акцепторном сайте сплайсинга пятого интрона гена GCK. Обнаруженный вариант сегрегировал с патологическим фенотипом у обследованных членов семьи. Ген GCK кодирует глюко- киназу (гексокиназу 4), которая катализирует первый шаг в различных путях метаболизма глюкозы. Мутации в этом гене ассоциированы с сахарным диабетом взрослого типа у молодых, подтип 2 (MODY2). Заболевание характеризуется незначительным повышением глюкозы натощак, хорошо контролируется медикамента- ми и отличается низкой распространенностью микро- и макрососудистых осложнений. Представленный в исследовании случай MODY2 выявил клиническую значимость мутации в сайте сплайсинга гена GCK. При возникновении у молодых людей и беременных женщин неклассического сахарного диабета проведение генетического тестирования необходимо для подтверждения диагноза и оптимального выбора тактики и способа лечения.

## Introduction

Maturity onset diabetes of the young (MODY) is a hereditary form of diabetes with autosomal dominant inheritance and is characterized by onset at a young age and by the presence of an initial defect in pancreatic β-cell function. This type of diabetes differs from classic types of diabetes mellitus-type 1 (DM1) and type 2 (DM2) in disease progression, in treatment strategies, and prognosis (Anık et al., 2015). Up to 80 % of MODY cases are not detected or are misdiagnosed as DM1 or DM2; therefore, patients with an incorrectly diagnosed type of diabetes are often prescribed inadequate therapy (Shields et al., 2010). On average, MODY is detected in 2–5 % of cases of diabetes (the rest being mostly DM1 and DM2) (Fajans et al., 2001). To reliably diagnose MODY in a patient, molecular genetic analysis should be carried out. To date, 14 types of MODY (MODY1 through MODY14) have been identified, each associated with mutations in a specific gene: HNF4A, GCK, HNF1A, PDX1, HNF1B, NEUROD1, KLF11, CEL, PAX4, INS, BLK, KCNJ11, ABCC8 and APPL1 (Thanabalasingham et al., 2011; Bonnefond et al., 2012; McDonald et al., 2013; Lachance, 2016; Ovsyannikova et al., 2016). Fourteen MODY-associated genes explain 70–85 % of the disease cases and are involved in various stages of glucose metabolism regulation (Thanabalasingham et al., 2011; Bonnefond et al., 2012; Lachance, 2016). According to various researchers, 11 to 30 % of MODY cases are caused by mutation in other genes (Edghill et al., 2010; Bonnefond et al., 2012). These forms of MODY are commonly referred to as MODY-X. Because the vast majority of pathogenic mutations are found in exons and adjacent splicing sites of genes (Stenson et al., 2017), it is reasonable to perform whole-exome sequencing on genomic DNA from individuals with MODY, with subsequent genetic testing of their relatives for the identified mutation. MODY verification allows for successful patient management and ensures healthy pregnancy and provision of genetic counseling to families (Lachance, 2016). Examination of relatives of MODY probands makes it possible to diagnose hyperglycemia in the preclinical phase.

In this report, we describe a clinical case of a family with MODY2 associated with a rare splice site mutation in the glucokinase (GCK ) gene identified by the next-generation sequencing technology.

## Materials and methods

The study protocol was approved by the local Ethics Committee of the Institute of Internal and Preventive Medicine (Branch of the Institute of Cytology and Genetics of Siberian Branch of the Russian Academy of Sciences, Novosibirsk, Russia, approval # 22.06.2008). Written informed consent to be examined and to participate in the study was obtained from each patient. For individuals younger than 18 years, the informed consent form was signed by a parent or legal guardian.

Blood samples were collected from the ulnar vein for biochemical analysis in the morning on an empty stomach. Lipid levels (cholesterol, triglycerides, low-density lipoprotein cholesterol, and high-density lipoprotein cholesterol) and glucose concentration were determined on a KoneLab 300i biochemical analyzer (Thermo Fisher Scientific, Waltham, MA, USA) with Thermo Fisher Scientific reagents.

Genomic DNA was isolated from leukocytes of venous blood by phenol-chloroform extraction (Sambrook, Russell, 2006). Quality of the extracted DNA was assessed on a capillary electrophoresis system, Agilent 2100 Bioanalyzer (Agilent Technologies Inc., USA). Sequencing of patients’ DNA was carried out on an Illumina HiSeq1500 instrument (Illumina, San Diego, CA, USA). The enrichment and library preparation were performed with the SureSelectXT Human All Exon V5 + UTRs Kit (Agilent Technologies Inc., USA). Reads were mapped to the reference human genome (GRCh37) by means of the Burrow–Wheeler Alignment tool (BWA v.0.7.12) (Li, Durbin, 2009). Polymerase chain reaction (PCR)-generated duplicates were removed in the PICARD software (https://broadinstitute.github.io/picard/).

A search for single-nucleotide variants (SNVs) was conducted using the Genome Analysis Toolkit v.3.3 package by the procedure for local remapping of short insertions/deletions and recalibration of read quality (McKenna et al., 2010). The depth of coverage was 34× to 53×. SNVs with genotype quality scores < 20 and coverage depth < 10× were filtered out and excluded from further analysis. Annotation of the SNVs was performed in the ANNOVAR software (Wang et al., 2010) using the 1000 Genomes Project (The 1000 Genomes Project Consortium…, 2015) and The Genome Aggregation Database (gnomAD) (Karczewski et al., 2019) databases. We selected the spectrum of rare and novel sequence variants in MODY genes (HNF4A, GCK, HNF1A, PDX1, HNF1B, NEUROD1, KLF11, CEL, PAX4, INS, BLK, KCNJ11, ABCC8 and APPL1). Rare variants were selected if their minor allele frequency (MAF) was ≤ 0.5 in the 1000 Genomes Project and gnomAD. Heterozygous substitution c.580–1G>A (IVS5 –1G>A) at an acceptor splice site of intron 5 of the GCK gene was found in the proband and her sister. To predict the possible effect of the SNV on splicing regulation, we employed the SPANR software (Xiong et al., 2015).

The substitution was corroborated by Sanger sequencing of the DNA fragment containing exons 5 and 6, intron 5, and parts of introns 4 and 6 using the following forward and reverse primers: 5′-CAGGGAGCCTCAGCAGTCTGGA-3′ and 5′-GCCACGAGGCCTATCTCTCCCC-3′. The oligonucleotides were designed in the Primer-Blast software (https:// www.ncbi.nlm.nih.gov/tools/primer-blast/) and were synthesized by the Biosset company (Russia, Novosibirsk). The sequencing reactions were carried out on an automated ABI 3500 DNA sequencer (Thermo Fisher Scientific, USA) with the BigDye Terminator v3.1 Cycle Sequencing Kit (Thermo Fisher Scientific, USA). PCR was set up using BioMaster LR HS-PCR (2×) (BioLabMix, Russia), 1 μL of each primer, and 1 μL of DNA, with a total final volume of 25 μL. The thermocycling program consisted of initial denaturation at 94 °C for 3 min and then 35 cycles at 94 °C for 30 s, 66 °C for 30 s, and 72 °C for 50 s. The PCR products were evaluated by electrophoresis in a 5 % polyacrylamide gel after visualization with an ethidium bromide solution. A 100 bp DNA Ladder (BioLabMix) was simultaneously run on the gel as molecular size markers. The amplicons were purified using Agencourt AMPure Xp beads (Beckman Coulter, USA). The sequencing reactions were conducted on an automated ABI 3500 DNA sequencer (Thermo Fisher Scientific, USA) via the BigDye Terminator v3.1 Cycle Sequencing Kit (Thermo Fisher Scientific). The sequences were analyzed in the Vector NTI® Advance software (Thermo Fisher Scientific). The hg19 version of the human genome served as a reference sequence for the alignment.

## Results

The white European 44-year-old female proband was under medical observation. When she underwent routine screening in 2012 (at age 40), hyperglycemia at 7.2 mmol/L was revealed. No complaints were registered. During subsequent glycemia control, maximal fasting glucose was 7.2 mmol/L, and the postprandial one was 8.9 mmol/L. C-peptide was 1.83 ng/mL (reference range 0.5–3.2 ng/mL), immunoreactive insulin was 7.9 μU/mL (reference range 2.0–25.0 μU/ mL), and glycated hemoglobin (HbA1c) was 7.1 %. Antibodies to insulin, to pancreatic islet cells, and to glutamic acid decarboxylase were absent. Blood biochemical analysis and determination of thyroid status did not reveal any abnormalities. Ultrasonography of internal organs, echocardiography, and a study of brachiocephalic vessels did not uncover any pathology. The body mass index (BMI) was 20.2 kg/m2. DM2 was diagnosed in the patient, and sitagliptin was prescribed. At the age of 26, the patient spontaneously delivered a healthy girl at 39 weeks of gestation; hyperglycemia was not detected during the pregnancy.

The sister of the proband is a white European 35-yearold woman. At age 23, during tests before mastectomy for mastopathy, she got a diagnosis of fasting hyperglycemia (6.3 mmol/L). The patient did not have any complains, and a proper diet was recommended. At the age of 29, during additional examination before cholecystectomy for cholelithiasis, she received a diagnosis of DM2, and vildagliptin was prescribed at the dose of 50 mg twice a day. At age 31, the proband’s sister visited an endocrinologist at the outpatient clinic of the Institute of Internal and Preventive Medicine (Branch of the Institute of Cytology and Genetics of Siberian Branch of the Russian Academy of Sciences, Novosibirsk, Russia) with complaints of a failure to get pregnant within a year. On examination, BMI was 20.6 kg/m2, and the objective status was unremarkable. Blood biochemical analysis revealed hypercalcemia (3.25 mmol/L), increased levels of high-density lipoprotein cholesterol (85 mg/dL), hypercholesterolemia (220 mg/ dL), and hyperglycemia (6.8 mmol/L), but other analyzed parameters were within reference ranges. The HbA1c level was 7.1 %. Antibodies to insulin, to pancreatic islet cells, and to glutamic acid decarboxylase were absent. Thyroid-stimulating hormone concentration was 0.759 mU/ mL (reference range 0.4–4.0), whereas the prolactin level was 216 ng/ mL (reference range 1.2–19.5). Echocardiography, Doppler sonography of extracranial parts of cerebral vessels, and abdominal and renal ultrasonographic examination revealed no pathology. Cysts were found in both thyroid lobes during the ultrasonography. Given the existence of the proband’s relatives with impaired glucose metabolism, persistence of normal C-peptide levels, the absence of diabetes-associated autoantibodies, normal BMIs of the proband and her sister, and stable mild hyperglycemia, MODY was assumed.

Exons and adjacent splice sites of MODY-associated genes were analyzed by whole-exome sequencing in the proband and her sister. As a result, heterozygous substitution c.580–1G>A (IVS5 –1G>A) at an acceptor splice site of intron 5 of the GCK gene was found in the proband and her sister. The IVS5 (–1G>A) polymorphism of GCK was submitted in ClinVar with an accession number of rs1554335421 (Landrum et al., 2018), but was absent in the 1000 Genomes Project (The 1000 Genomes Project Consortium…, 2015), in gnomAD project databases at the moment of publication. Subsequent genetic analysis by Sanger sequencing of the family members (mother, father, daughter, and nephew of the proband) uncovered segregation of the substitution with DM as an autosomal dominant trait (see the Figure).

**Fig. 1.**
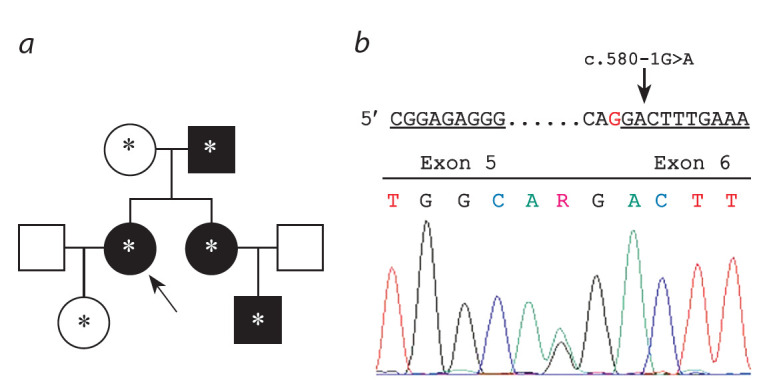
Mutation identified in GCK gene. a – family history with inherited diabetes mellitus (DM). Asterisk indicates medically examined family members; b – schematic representation of the mutation in the splicing acceptor site and chromatogram of DNA sequence with mutated allele c.580 –1G>A (IVS5 –1G>A, rs1554335421) of the GCK gene.

Our results, literature data, and databases suggest that this splice site mutation is likely pathogenic.

After confirmation of GCK-MODY in the proband and her sister, their relatives were screened for carbohydrate metabo

lism disorders. The proband’s mother and daughter did not have any abnormalities. The proband’s father showed impaired fasting glucose. No complaints were registered, venous plasma fasting glucose was 6.3 mmol/L, and 2 h after the oral glucose tolerance test, it was 7.5 mmol/L. At present, the man does not take any medication. The same heterozygous substitution rs1554335421 (IVS5 –1G>A) in the proband’s father’s GCK gene was detected by genetic testing.

The proband’s sister had her first pregnancy in 2014 (at age 31). In 2015, a boy weighing 3640 g was born by a caesarean section at 39 weeks of gestation. The pregnancy was complicated: premature rupture of membranes, weakness of labor, and fetal hypoxia. After delivery, due to stable glycemic indexes, it was decided that insulin therapy should be discontinued. In January 2018, during treatment with diet, the patient’s HbA1c was 6.4 %.

The neonatal period of the proband’s nephew was unremarkable. In 2017, his blood biochemical analysis resulted in a diagnosis of hyperglycemia (6.9 mmol/L). HbA1c was 6.3 %, and the C-peptide level was 0.54 ng/mL. Antibodies to insulin, pancreatic β-cells, and glutamic acid decarboxylase were undetectable. The same heterozygous substitution rs1554335421 (IVS5; –1G>A) in the GCK gene was identified by genetic testing. At present, the child is under medical observation at Almazov Federal Medical Research Centre (Saint Petersburg, Russia); because of GCK-MODY, a balanced diet was recommended.

**Discussion**

It is known that in young patients with impaired carbohydrate metabolism, DM1, DM2, or rarer monogenic forms of diabetes may be diagnosed. At the onset of the disease, the proband and her sister had no symptoms characteristic for the common types of diabetes, fasting hyperglycemia was not progressing, and carbohydrate metabolism disorders were detected during routine screening. The presence of DM in the proband’s sister, persistence of normal C-peptide levels, a lack of autoantibodies, and a normal BMI in the proband and her sister pointed to MODY (Chakera et al., 2015).

Heterozygous splice site mutation c.580–1G>A (rs1554335421) in intron 5 of their GCK gene was identified by genetic testing. Mutations in this gene are associated with DM2, MODY, and neonatal DM (Plengvidhya et al., 2009; Lachance, 2016). More than 600 variants of the GCK gene associated with MODY have been described, and the list of the mutations is constantly growing. The vast majority of the mutations are missense substitutions, but splice site mutations, deletions, and insertions are reported too (Stenson et al., 2017).

The GCK gene is located in chromosomal region 7p15.3- p15.1 and consists of 12 exons that encode a 465-amino-acid protein, glucokinase (Osbak et al., 2009), which is one of four members of the hexokinase family of enzymes. In 1992, GCK was the first gene to be linked to MODY. It plays an important regulatory role in glucose metabolism. Glucokinase catalyzes phosphorylation of glucose to produce glucose-6-phosphate as the first step of glycolysis in pancreatic β-cells (Matschinsky et al., 1993; Iynedjian, 2009). Most individuals with heterozygous GCK mutations show fasting plasma glucose levels between 5.5 and 8.0 mmol/L and a small increase in plasma glucose (< 3 mmol/L in 70 % of the patients) 2 h after the oral glucose test (Stride et al., 2002). This feature also explains asymptomatic fasting hyperglycemia (HbA1c range 5.8–7.6 % (40–60 mmol/mol)) and rare microvascular and macrovascular complications in patients with GCK-MODY (Caetano et al., 2012; Steele et al., 2014). Most patients have an aberrant fasting glucose level or impaired glucose tolerance, and less than 50 % of the affected individuals have diabetes, which is diagnosed during childhood, adolescence, or pregnancy (Caetano et al., 2012). In a study on Italian patients under 18 years of age with incidental hyperglycemia, it was estimated that 15 % of these cases are caused by GCK mutations (Lorini et al., 2009).

It was found here that the proband and her sister carry a heterozygous substitution, c.580–1G>A (IVS5 –1G>A, rs1554335421), at an acceptor splice site of GCK intron 5. This allelic variant is of interest because consensus donor (GT dinucleotide) and acceptor (AG dinucleotide) splice sites are highly conserved. Point mutations at these loci can lead to cryptic splice site activation and synthesis of aberrant protein isoforms.

In silico analysis of the functional significance of this substitution suggested that the inclusion of exons 5, 6, and 7 in gene transcripts will be reduced in case of the detected variant (see the Table).

**Table 1.**
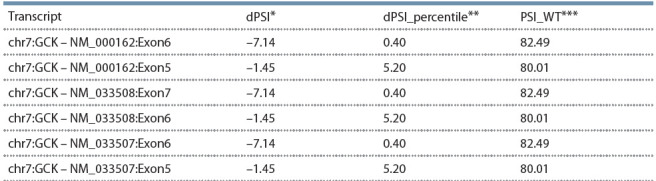
The predicted (by SPANR) effect of c.580 –1G>A (IVS5 –1G>A, rs1554335421) of the GCK gene on splicing Note. PSI – percentage of transcripts with the exon spliced in: * dPSI: a maximal difference in PSI across 16 tissues; ** dPSI_percentile: percentile of mutant dPSI among dPSIs of common SNPs; *** PSI_WT: predicted PSI in the wild type.

DM and a family history of gestational diabetes. Experiments on lymphoblastoid cells indicate that rs1554335421 (IVS5 –1G>A) of GCK can activate a cryptic splice site in intron 5 and cause retention of 27 bp of the intron (Toaima et al., 2005). Information about rs1554335421 (IVS5 –1G>A) of GCK is absent in the 1000 Genomes Project, The Exome Aggregation Consortium, and GNOMAD (https://gnomad. broadinstitute.org/) databases; however, taking into account the previous study and the data we obtained here, carriage of the A allele at position –1 of intron 5 is most likely a causative dominant variant of the GCK gene in MODY-affected people.

Cryptic splice site activation and formation of several alternative transcripts with intron 7 fragments’ retention were demonstrated in a system of model GCK minigenes with acceptor site mutation IVS7 (–1G>C) (Igudin et al., 2014).

Model mice with the homozygous mutation in the splice site of β-cell-speciﬁc exon 1 IVS1A (–1G>T) show hyperglycemia, glucosuria, and growth retardation and die within the ﬁrst week after birth. This phenotype can be explained by exon skipping or intron retention (Inoue et al., 2004). Splicing sites affected by mutations have been described for many pathological phenotypes: neurofibromatosis type 1 (Jang et al., 2016), familial hypercholesterolemia (Shakhtshneider et al., 2017), Wiskott–Aldrich syndrome and chronic colitis (Esmaeilzadeh et al., 2018), hypophosphatemic rickets (Ma et al., 2015), and others. Mutations affecting splicing have been found not only in canonical splicing sites but also in introns and exons and may have a tissue-specific effect, as in familial dysautonomia (Slaugenhaupt et al., 2001; Abramowicz, Gos, 2018). That analysis indicated that the donor splice site mutations were more prevalent than the acceptor splice site variants (ratio 1.5:1.0) (Abramowicz, Gos, 2018). Because the mutations in the GCK gene can cause a mild clinical phenotype, which can vary under the influence of many genetic and lifestyle factors, research on the carriers of these mutations is essential for identifying additional risk factors.

GCK-MODY is inherited as an autosomal dominant trait manifested throughout the lifespan as stable, mild fasting hyperglycemia usually reaching 6.7 mmol/L and higher only in middle age (Wedrychowicz et al., 2017). A similar pattern was observed here in the proband and her sister. Nonetheless, the metabolic disturbances in the carriers of GCK mutations are present from birth and can be identified already in the first years of life, almost all of them after puberty (Steele et al., 2014). The proband’s nephew, who is a heterozygous mutation carrier, developed carbohydrate metabolism disorders at two years of age.

It has been reported that carriers of GCK gene mutations with a long history of hyperglycemia (48.6 years on average) usually have micro- and macrovascular complications of diabetes and are at a risk of cardiovascular diseases that is identical to that in the general population (Pruhova et al., 2013).

Patients with GCK-MODY in childhood and adolescence can be treated only with diet in most cases, and glucoselowering therapy should be considered during pregnancy (Lachance, 2016). The present subtype of MODY2 (in the proband and her sister) currently is treated with diet resulting in sufficient glycemic control.

The presence or absence of a GCK mutation in the fetus affects its sensitivity to maternal hyperglycemia (Chakera et al., 2012). If the fetus does not have the mutation, then it will secrete insulin excessively and as a result have a risk of macrosomia (Spyer et al., 2009). In that case, low doses of insulin should be prescribed during pregnancy (Chakera et al., 2014). During her pregnancy, the proband’s sister was given insulin injections in small doses. After delivery, insulin therapy was discontinued. Subsequently, the child was found to carry the same substitution.

## Conclusion

The presented subtype of MODY2 reveals the clinical significance of the mutation in a splice site of the GCK gene. When nonclassical diabetes mellitus is being diagnosed in young people and pregnant women, genetic testing is needed to verify the diagnosis and to select the optimal treatment method.

## Conflict of interest

The authors declare no conflict of interest.
